# Identifying clustering in patterns of late effects among survivors of adolescent and young adult Hodgkin lymphoma

**DOI:** 10.1093/jncics/pkaf094

**Published:** 2025-10-01

**Authors:** Kellee Parker, Mallorie B Heneghan, Qian W Li, Ann Brunson, Judy Ou, Heydon K Kaddas, Renata Abrahão, Jessica Chubak, Karen J Wernli, Brad Zebrack, Erin E Hahn, Lawrence H Kushi, Hazel B Nichols, Theresa Keegan, Anne C Kirchhoff

**Affiliations:** Department of Pediatrics, University of Utah, Salt Lake City, UT, United States; Huntsman Cancer Institute, University of Utah, Salt Lake City, UT, United States; Department of Pediatrics, University of Utah, Salt Lake City, UT, United States; Huntsman Cancer Institute, University of Utah, Salt Lake City, UT, United States; University of California, Davis Comprehensive Cancer Center, Sacramento, CA, United States; University of California, Davis Comprehensive Cancer Center, Sacramento, CA, United States; Department of Pediatrics, University of Utah, Salt Lake City, UT, United States; Huntsman Cancer Institute, University of Utah, Salt Lake City, UT, United States; Huntsman Cancer Institute, University of Utah, Salt Lake City, UT, United States; University of California, Davis Comprehensive Cancer Center, Sacramento, CA, United States; Kaiser Permanente Washington Health Research Institute, Seattle, WA, United States; Kaiser Permanente Washington Health Research Institute, Seattle, WA, United States; School of Social Work, University of Michigan, Ann Arbor, MI, United States; Department of Research and Evaluation, Kaiser Permanente Southern California, Pasadena, CA, United States; Division of Research, Kaiser Permanente Northern California, Pleasanton, CA, United States; Department of Epidemiology, University of North Carolina Gillings School of Global Public Health, Chapel Hill, NC, United States; University of California, Davis Comprehensive Cancer Center, Sacramento, CA, United States; Department of Pediatrics, University of Utah, Salt Lake City, UT, United States; Huntsman Cancer Institute, University of Utah, Salt Lake City, UT, United States

## Abstract

**Background:**

We examined late effects clustering among adolescent and young adult (AYA; age 15-39 years at diagnosis) Hodgkin lymphoma (HL) survivors and identified characteristics associated with each cluster.

**Methods:**

We included AYAs with HL in 2006-2018 from the California and Utah Cancer Registries linked to statewide hospitalization, emergency department, and ambulatory surgery visit data. We identified severe late effects >2 years after cancer diagnosis in 9 late effects categories. Latent class analysis (LCA) was used to identify late effects clusters. Multinomial logistic regression models estimated adjusted associations of demographic and treatment characteristics with LCA late effect group.

**Results:**

We identified 4635 AYA HL survivors with median follow-up of 8.2 years and 4 late effects groups: 77.1% had a low probability of any late effect (Low Morbidity), 12.8% had high probability of Thyroid disorders, 8.0% had high probability of Cardiovascular Disease (CVD), and 2.1% had high probability of Multiple Conditions (CVD, diabetes/pancreatic, thyroid, and renal diseases). Publicly insured AYAs were more likely than those with private insurance to be in the CVD (OR = 1.53, 95% CI = 1.18 to 1.98) and Multiple Conditions (OR = 2.17, 95% CI = 1.29 to 3.66) than the Low Morbidity group. AYAs with radiation were more likely to be in the Multiple Conditions (OR = 2.31, 95% CI = 1.41 to 3.78) and Thyroid (OR = 2.81, 95% CI = 2.20 to 3.58) groups. Hematopoietic cell transplantation was associated with Multiple Conditions (OR = 9.50, 95% CI = 5.82 to 15.50), CVD (OR = 3.82, 95% CI = 2.96 to 4.93), and Thyroid (OR = 2.86, 95% CI = 2.12 to 3.85) groups.

**Conclusions:**

While most AYA HL survivors were in the Low Morbidity group, those with public insurance or intense treatment may be at higher risk for multiple conditions.

## Background

Classic Hodgkin lymphoma (cHL) is one of the most common types of cancer diagnosed among adolescents and young adults (AYA) aged 15 to 39 years old.^1^ Five-year overall survival has surpassed 85% for cHL, resulting in a growing number of patients who are long-term survivors.^2^ While cHL is highly curable, successful treatment regimens require multiagent chemotherapy, with or without radiation, putting survivors at risk for late effects including major cardiovascular events, respiratory disease, chronic kidney disease/renal failure, chronic liver disease, endocrine disease, avascular necrosis, and secondary malignant neoplasms (SMN).^3^ Given the young age at diagnosis, most survivors of AYA cHL will go on to live many more decades, which makes minimizing late effects critical for this patient population.^3-5^

Studies of cHL from the Childhood Cancer Survivor Study (CCSS) demonstrated a substantial risk of late effects in pediatric cHL survivors,^4^^,^^5^ but the findings may not extend to the AYA cHL population for several reasons. These include differences in treatment location (pediatric vs adult, community vs academic medical center) that may impact the robustness of long-term follow-up care, choice of treatment, and physiologic changes related to aging that may impact treatment tolerance.^2^ Treatment of pediatric cHL happens almost exclusively by pediatric oncologists within academic medical systems, whereas AYA cHL care is dispersed across adult and pediatric care centers and across academic and community sites.^6^^,^^7^ Data from Utah found 12% of AYAs with lymphoma are treated by academic pediatric oncologists, 26% are treated at an adult academic facility, and the remaining are treated by adult community providers.^8^ Reports from other states show similar patterns.^9^

While recent efforts including the cross-National Clinical Trials Network collaboration seek to standardize care for AYAs, there is currently no consensus-based treatment approach globally for this age group, and AYAs with cHL may be treated on adult or pediatric treatment protocols.^2^ Historically, cHL pediatric treatment protocols have included dose dense chemotherapy regimens that seek to limit cumulative anthracycline and alkylating exposure in attempts to maintain high event-free survival while minimizing late effects.^2^ Furthermore, pediatric protocols are more likely to utilize radiation than adult protocols, but at lower doses.^10^

Previous studies in AYAs have examined late effects as separate outcomes, without considering that multiple late effects may be biologically linked.^3^ In addition, the development of one late effect may accelerate the onset of or exacerbate the severity of another (eg diabetes and cardiovascular disease).^11^^,^^12^ Identifying whether late effects cluster among AYA cHL survivors and how these clusters are associated with clinical and sociodemographic factors could provide valuable and unique information to guide surveillance and management for late effects. Additionally, it may also be important to identify survivors at increased risk of long-term and co-occurring late effects, as healthcare costs associated with late effects can be high.^13^

To address these gaps in research, we report on findings from the “Valuing Opinions and Insight from Cancer Experiences” (VOICE) study. VOICE represents a recently treated cohort (2006-2018) from a time when risk-adapted treatment protocols were widely utilized in clinical practice.^14^ Using VOICE, we estimated the incidence of severe late effects among AYA cHL survivors and described patterns of late effects among AYA cHL survivors. We also examined how risks vary by initial treatment approaches, race/ethnicity, neighborhood socioeconomic status, and health insurance status, among other key patient factors.

## Methods

### Data overview

The VOICE Study^14^ includes AYA cancer survivors identified from several data sources, including the Kaiser Permanente Northern California and Kaiser Permanente Southern California healthcare systems, the North Carolina Cancer Information Population Health Resource and linkages of statewide cancer registry, and healthcare utilization data from the states of California and Utah. The analyses presented here focus on AYA cancer survivors from statewide linkages between cancer registries and healthcare utilization data in California and Utah.

We identified AYAs diagnosed with cHL from the California Cancer Registry (CCR) and the Utah Cancer Registry (UCR), which is part of the Utah Population Database (UPDB). UCR and CCR are members of the National Cancer Institute (NCI) Surveillance, Epidemiology, and End Results (SEER) program, which tracks cancer diagnosis and summary treatment information for all persons diagnosed or treated for cancer in their geographic coverage areas. CCR is linked to the California Department of Health Care Access and Information (HCAI) data on hospitalizations, emergency room visits, and ambulatory surgery visits for the entire state. UPDB links UCR to statewide health claims administrative records for the Utah population and includes statewide data on hospitalization, emergency room visits, and ambulatory surgery visits. For both states, statewide inpatient hospitalization data includes a record for each inpatient discharged from any non-federal acute care hospital. This study was approved by the Institutional Review Boards at the University of Utah, the Utah Resource for Genetic and Epidemiologic Research, the California Committee for the Protection of Human Subjects, and Kaiser Permanente Northern California Institutional Review Board.

### Cohort selection and follow-up

We identified 5843 people diagnosed with a first primary cHL at age 15 to 39 years between 2006 and 2018 while living in California or Utah. As our focus was on late effects, we limited this population to cHL survivors with ≥2 years of follow-up after diagnosis (*n* = 636 were excluded) who could be linked to health claims data from HCAI or the UPDB (*n* = 559 with unknown or invalid SSN were excluded). Additionally, patients with a diagnosis of HIV/AIDS or a concurrent cancer diagnosis within 60 days of their cHL cancer diagnosis (*n* = 9) were excluded. We followed 4635 (total analysis cohort) survivors from 2 years after first cHL diagnosis to death, date of known last vital status, or end of study as of December 31, 2020, whichever occurred first (Figure S1).

### Late effects ascertainment

Using HCAI and UPDB inpatient hospitalization, emergency department, and ambulatory surgery data, late effects were identified as health conditions of interest that were diagnosed >2 years after cHL diagnosis. Late effects included the following diseases: cardiovascular, diabetes/pancreatic, thyroid, liver, renal, respiratory, avascular necrosis, venous thromboembolism, and SMN. The use of hospitalization, emergency department, and ambulatory surgery data to identify these late effects indicates that these health conditions were severe enough to require care in these settings. The exception to this were SMN, which were identified from the CCR and UCR beginning 60 days after diagnosis of cHL, as done in prior studies of SMN.^15^ We ascertained late effects by International Classification of Diseases, Ninth or Tenth Revision, Clinical Modification (ICD-9-CM/ICD-10-CM) diagnosis codes and Current Procedure Terminology (CPT) procedure codes, and flagged individuals with at least one ICD-9/ICD-10 code that fell into each disease category (Yes/No). To reduce the likelihood of misclassifying pre-existing conditions as late effects, any conditions identified in the 5 years prior to cHL diagnosis were not included as outcomes in our analyses but those survivors still remained in the cohort and development of new late effects was included in analysis. Conditions diagnosed initially within 2 years after cancer diagnosis had to persist after the index date to be considered as an outcome in this analysis (SMN occurring within 2 years were considered as prevalent outcomes at the beginning of study follow-up). The 2-year threshold was selected to distinguish late effects from acute or treatment-related toxicities, which are more likely to occur during or shortly after therapy, and is similar to methods commonly used in survivorship research.^16^^,^^17^

### Demographics, cancer, and treatment data

Sex, race, ethnicity, age at diagnosis, year of diagnosis, stage at diagnosis, health insurance status at first cancer diagnosis, and deaths and year of death were provided by the cancer registries. Registries classified race and ethnicity into mutually exclusive categories as Non-Hispanic White, Non-Hispanic Black, Hispanic, Asian, Pacific Islander, American Indian, and unknown.

Neighborhood socioeconomic status (SES) was ascertained based on residential address at the date of cHL diagnosis.^18^ The Yost index is a composite measure of SES that combines 7 components, including education index, proportion with a blue-collar job, proportion older than 16 years in the workforce without a job, median household income, proportion below 200% of the poverty level, median rent, and median house value.^15^ Neighborhoods were classified into highest, middle, and lowest SES by tertile based on the distribution in each state of residence.

Cancer variables included American Joint Committee on Cancer (AJCC) stage at diagnosis (I–IV) and histologic subtype (nodular sclerosis, mixed cellularity, lymphocyte depleted, lymphocyte rich, and classic HL NOS). Cancer treatment was ascertained using registry data for chemotherapy and radiation therapy, and for hematopoietic cell transplantation (HCT) data from the cancer registry, HCAI, and UPDB.

### Statistical methods

Descriptive statistics summarized the sociodemographic and clinical characteristics of the cohort. The cumulative incidence and associated 95% confidence intervals (CIs) of developing a late effect were calculated using nonparametric methods that account for death as a competing risk.^19^

We utilized latent class analysis (LCA) to determine whether patterns of late effects among the 9 disease categories could be identified as discrete, mutually exclusive population classes. Binary indicators of late effects were used in the LCA models. An iterative approach was used to identify the number of latent classes that best fit the model according to the lowest Akaike information criterion (AIC), G^2^ value, contextual contribution of the classes, and clinical relevance.^20^^,^^21^ Class membership reflects the most probable pattern of co-occurring conditions using the healthcare data and observed time periods. The model also provided posterior probabilities of each disease category, which were used to assign a class to each survivor. Using the posterior probabilities derived from LCA to assess potential misclassification, we observed that 60 patients (1.3%) had a final posterior probability of less than 0.5, while 297 patients (6.4%) had a final posterior probability of less than 0.6, resulting in an overall classification accuracy of 92.3%.

Distributions of late effects were plotted according to the groups identified in the LCA. This was done to determine if the LCA classes represented distinct groups with distributions of disease (non-parallel lines) or if they represented a single group that was artificially split into multiple groups representing varying degrees of disease (parallel lines) (Figure S2).^22^ A sensitivity analysis was performed comparing survivors with less than 5 years and more than 5 years of follow-up to determine if differences in the latent classes were due to the time frame of measuring the outcomes.

Multivariable-adjusted, multinomial logistic regression was used to estimate the association of sociodemographic characteristics, cancer stage at diagnosis, and treatment with membership within the assigned LCA group. In addition, Cox proportional hazards regression models were used to calculate adjusted hazard ratios (HR) and 95% CIs for the associations of each late effect with sociodemographic and clinical characteristics. For both the multinomial logistic regression and Cox regression, we included age at diagnosis, sex, race/ethnicity, health insurance status at diagnosis, neighborhood SES, stage at diagnosis, radiation therapy, and HCT in the models. HCT was considered a time-varying variable in the models. For the Cox regression model with diabetes/pancreatic disease as the outcome, sex violated the proportional hazards assumption and was included as a stratifying variable in the model. We controlled for year of diagnosis to account for variation in diagnosis and therapies over time and state (CA/UT) to control for state-based differences in health insurance coverage, access to care, and other healthcare differences.

## Results

There were 4635 AYA cHL survivors included in the cohort with a median follow-up time of 8.24 years (interquartile range 5.09-11.44 years). Most survivors were diagnosed from ages 20-29 years (51.1%), were non-Hispanic White (55.8%), and had private or military insurance (71.9%; Table 1). The majority had early-stage disease (50.6% Stage II and 9.3% Stage I) and were treated with chemotherapy with radiation (32.7%) or chemotherapy without radiation (57.1%). Only 13.2% of patients had HCT. Overall, 95.9% of the cohort was alive at last follow-up; underlying cancer was the most common cause of death. The majority of survivors did not have any late effects (77.4%); 14.7% had 1 late effect, and 7.9% had 2 or more late effects (*data not shown*).

**Table 1.** Characteristics of 2-year survivors of adolescent and young adult Hodgkin lymphoma, Utah and California, 2006-2018.

*n* = 4635	No.	%
Age at diagnosis, years		
15-19	753	16.2
20-29	2368	51.1
30-39	1514	32.7
Sex		
Female	2335	50.4
Male	2300	49.6
Race/ethnicity		
American Indian	18	0.4
Asian	379	8.2
Hispanic	1279	27.6
Non-Hispanic Black	293	6.3
Non-Hispanic White	2588	55.8
Pacific Islander	22	0.5
Unknown	56	1.2
Health insurance at diagnosis/initial treatment		
Private/military	3334	71.9
Public	1059	22.8
Unknown	242	5.2
Neighborhood socioeconomic status (SES) at diagnosis		
Highest	1591	34.3
Lowest	1254	27.1
Middle	1716	37.0
Unknown	74	1.6
Year at diagnosis		
2006-2008	1274	27.5
2009-2011	1154	24.9
2012-2014	1046	22.6
2015-2018	1161	25.0
Stage at diagnosis		
Stage I	429	9.3
Stage II	2345	50.6
Stage III	873	18.8
Stage IV	741	16.0
Not applicable/unknown	247	5.3
Subtype		
Nodular sclerosis	3325	71.7
Mixed cellularity classical Hodgkin lymphoma	328	7.1
Lymphocyte-rich classical Hodgkin lymphoma	80	1.7
Classical Hodgkin lymphoma not otherwise specified (NOS)	902	19.5
Initial treatment		
Chemotherapy and radiation	1517	32.7
Chemotherapy only	2648	57.1
Radiation only	52	1.1
None/unknown	418	9.0
Hematopoietic cell transplantation		
Yes	612	13.2
No	4023	86.8
State		
California	4107	88.6
Utah	528	11.4
Vital status		
Alive	4447	95.9
Death from Hodgkin lymphoma	108	2.3
Death from other cause	80	1.8

### Patterns of late effects

**Figure 1.**
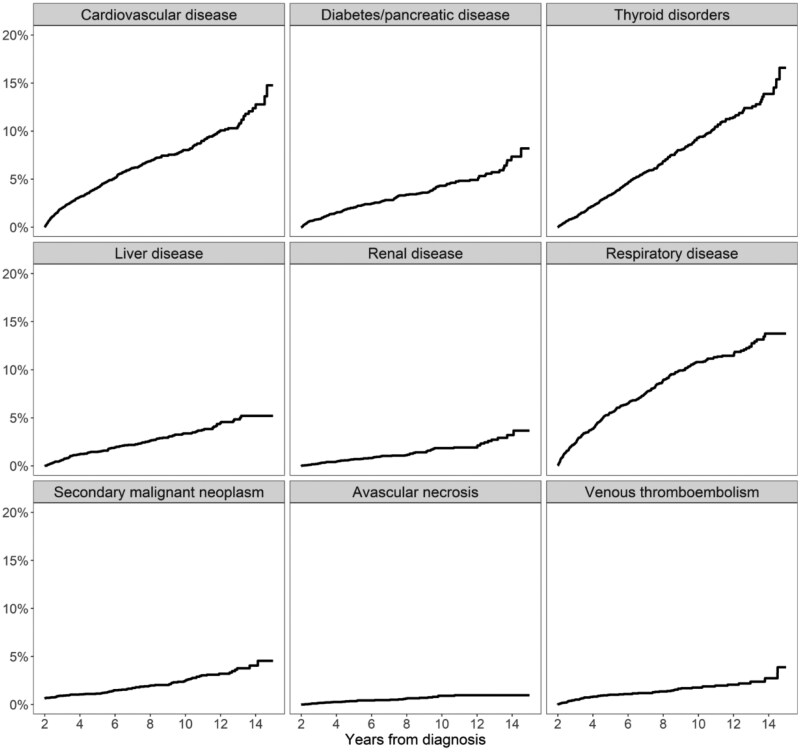
Cumulative incidence of each late effect among 2-year survivors of adolescent and young adult Hodgkin lymphoma. Secondary malignant neoplasm diagnosed between 60 days and 2 years of the first primary were considered prevalent at the time of entry into the analysis.

From years 2-10 after diagnosis, cumulative incidence was highest for cardiovascular (4.1% at 5 years, 95% CI = 3.6 to 4.8; 8.0% at 10 years, 95% CI = 7.1 to 9.0), thyroid (3.4% at 5 years, 95% CI = 2.8 to 3.9; 9.3% at 10 years, 95% CI = 8.3 to 10.4), and respiratory (5.5% at 5 years; 95% CI = 4.9 to 6.3; 10.8% at 10 years, 95% CI = 9.8 to 11.9) late effects (Figure 1; Table S1). The LCA analysis showed 4 distinct groups of late effects. A large majority of cHL survivors (77.1%) were estimated to have a low probability of late effects (Low Morbidity group; Table 2). Of the other 3 groups, 12.8% displayed an increased but low to moderate probability of thyroid disorders (0.40; Thyroid group) and 8% had a moderate probability of cardiovascular disease (0.41; Cardiovascular group). Both the Thyroid and Cardiovascular groups had lower probabilities of other conditions. However, a small group (2.1%) of survivors had increased likelihood of multiple health conditions (Multiple Conditions group), including cardiovascular disease (0.77), respiratory disease (0.72), as well as diabetes/pancreatic disease (0.53) and thyroid disease (0.53).

**Table 2.** Latent class analysis patterns in late effects among 2-year survivors of adolescent and young adult Hodgkin lymphoma.

	Low morbidity group	Thyroid group	Cardiovascular group	Multiple conditions group
Percentages estimated from LCA ^a^	77.0%	13.0%	8.0%	2.1%
Conditional probabilities from LCA ^b^				
Cardiovascular disease	0.01	0.12	0.41	0.77
Diabetes/pancreatic disease	0.01	0.04	0.15	0.53
Thyroid disorders	0.01	0.40	0.02	0.53
Liver disease	0.00	0.05	0.19	0.33
Renal disease	0.00	0.01	0.09	0.25
Respiratory disease	0.04	0.20	0.22	0.72
Secondary malignant neoplasm	0.01	0.07	0.06	0.17
Avascular necrosis	0.00	0.01	0.01	0.14
Venous thromboembolism	0.00	0.03	0.08	0.07
Median follow-up in years (IQR)	7.93 (4.95-11.18)	10.9 (8.21-13.02)	8.68 (5.05-11.9)	11.00 (7.95-12.35)

Abbreviation: LCA = latent class analysis.

a Calculated percentages of individuals in the data set that are predicted to belong to each identified latent class.

b Conditional probabilities of each late effect for members of the group.

**Figure 2.**
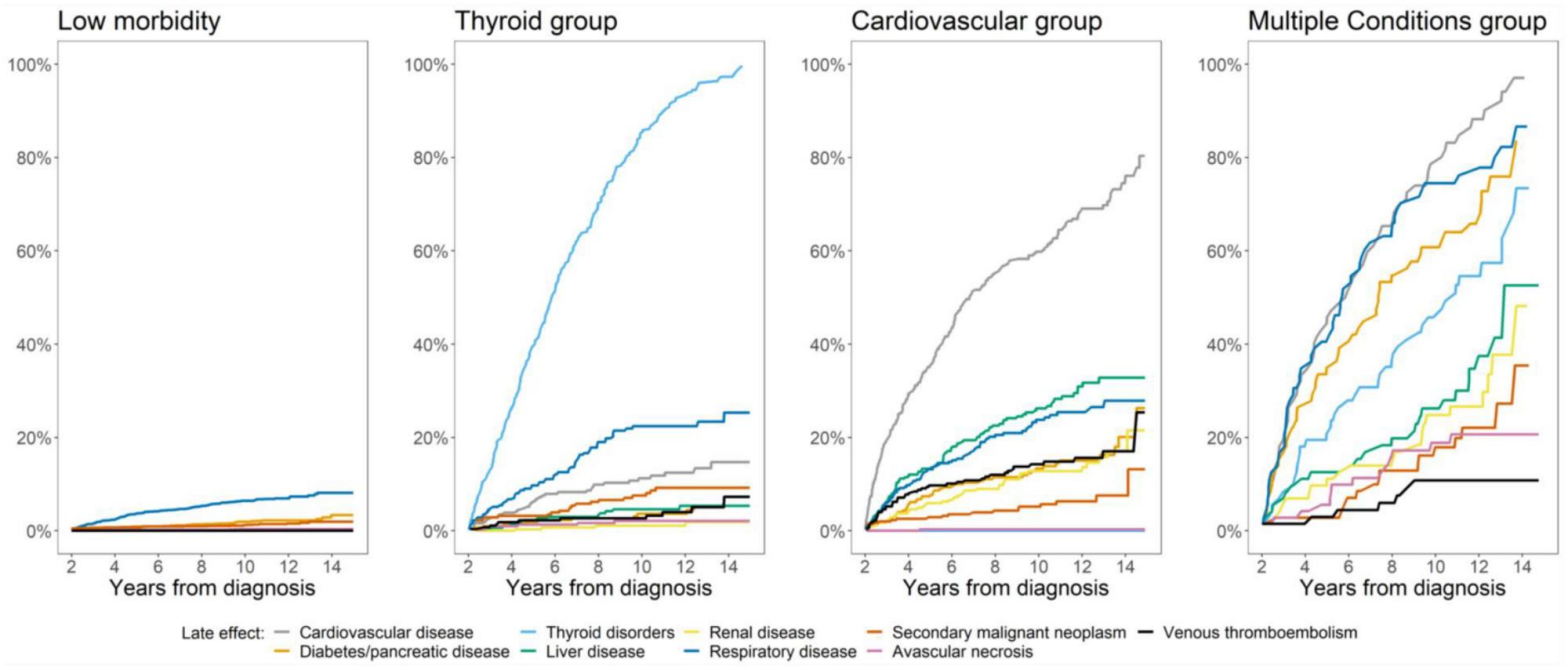
Cumulative incidence of each late effect by latent class analysis group in 2-year survivors of adolescent and young adult Hodgkin lymphoma.

We estimated cumulative incidence of late effects by the 4 LCA groups (Figure 2). In the Low Morbidity group, each late effect had an incidence of less than 7% at 10 years after cancer diagnosis. In the Cardiovascular group, the cumulative incidence of cardiovascular disease was 60% 10 years after cancer diagnosis with cumulative incidence of the other diseases less than 30% at this time; 85% of the Thyroid group had thyroid disorders by 10 years after cancer diagnosis with less than 25% for each other late effect. The Multiple Conditions group, by 10 years after cancer diagnosis, had a cardiovascular, respiratory, diabetes/pancreatic, and thyroid disorder incidence above or approaching 50%. Median duration of follow-up in the groups ranged from 7.98 years in the Low Morbidity group to 11.02 years in the Multiple Conditions group (Table 2). In the sensitivity analysis. there was no difference in clustering of late effects when we compared survivors with less and more than 5 years of follow-up.

### Multivariable LCA regression models

#### Sociodemographic factors

Survivors aged 15-19 years at diagnosis had lower odds of being in the Multiple Conditions group (odds ratio [OR] 0.36, 95% confidence interval [CI] 0.16 to 0.81) or Cardiovascular group (OR = 0.39, 95% CI = 0.26 to 0.58) than the Low Morbidity group compared to survivors aged 30-39 at diagnosis (Table 3). Additionally, survivors aged 20-29 years old at diagnosis had lower odds than those diagnosed at ages 30-39 years for being in the Cardiovascular group (OR = 0.73, 95% CI = 0.57 to 0.92). Female survivors had higher odds of belonging to the Thyroid group compared to male survivors (OR = 2.75, 95% CI = 2.12 to 3.57). Only non-Hispanic Black survivors had higher odds of being in the Cardiovascular group (OR = 1.84, 95% CI = 1.24 to 2.73) compared to non-Hispanic White survivors. Survivors with public insurance had higher odds of being in the Cardiovascular (OR = 1.53, 95% CI = 1.18 to 1.98) and Multiple Conditions (OR = 2.17, 95% CI = 1.29 to 3.66) groups than survivors with private/military insurance. Neighborhood SES was not significantly associated with any of the LCA groups compared to the Low Morbidity group.

**Table 3.** Multinomial logistic regression to estimate associations between sociodemographic and clinical factors and latent class analysis group classification among 2-year adolescent and young adult Hodgkin lymphoma survivors.

	Latent class analysis group (Reference = Low morbidity)		
	Cardiovascular	Thyroid disorders	Multiple conditions
	*n* = 353 (7.62%)	*n *= 313 (6.77%)	*n *= 73 (1.55%)
	OR (95% CIs)	OR (95% CIs)	OR (95% CIs)
Sociodemographic factors			
Age at diagnosis, year			
15-19	**0.39 (0.27 to 0.58)**	0.74 (0.52 to 1.07)	**0.35 (0.16 to 0.79)**
20-29	**0.73 (0.58 to 0.93)**	0.94 (0.72 to 1.23)	0.63 (0.38 to 1.05)
30-39	Reference	Reference	Reference
Sex			
Female	0.87 (0.69 to 1.09)	**2.80 (2.16 to 3.63)**	1.31 (0.81 to 2.11)
Male	Reference	Reference	Reference
Race/ethnicity			
Asian/Pacific Islander	1.74 (0.39 to 7.87)	0.74 (0.09 to 5.84)	3.24 (0.37 to 28.4)
Hispanic	1.13 (0.87 to 1.47)	1.16 (0.88 to 1.53)	0.72 (0.40 to 1.29)
Non-Hispanic Black	**1.74 (1.18 to 2.57)**	0.92 (0.53 to 1.58)	1.27 (0.55 to 2.96)
Non-Hispanic White	Reference	Reference	Reference
Health insurance			
Private/military	Reference	Reference	Reference
Public	**1.50 (1.16 to 1.94)**	1.01 (0.75 to 1.36)	**2.35 (1.40 to 3.94)**
Neighborhood socioeconomic status (SES)			
Lowest	Reference	Reference	Reference
Middle	0.82 (0.63 to 1.08)	0.86 (0.64 to 1.17)	0.72 (0.40 to 1.30)
Highest	0.73 (0.54 to 1.00)	0.87 (0.63 to 1.20)	0.65 (0.34 to 1.24)
Clinical factors			
Stage at diagnosis (AJCC)			
Stage I	Reference	Reference	Reference
Stage II	1.25 (0.86 to 1.81)	0.93 (0.66 to 1.31)	1.37 (0.57 to 3.33)
Stage III	1.42 (0.94 to 2.15)	0.90 (0.59 to 1.38)	1.49 (0.56 to 3.92)
Stage IV	1.51 (0.99 to 2.29)	0.72 (0.45 to 1.15)	1.28 (0.47 to 3.50)
Radiation			
Yes	1.18 (0.93 to 1.51)	**2.84 (2.23 to 3.63)**	**2.23 (1.37 to 3.64)**
No/unknown	Reference	Reference	Reference
Hematopoietic cell transplantation (HCT)			
Yes	**2.93 (2.24 to 3.84)**	**2.54 (1.86 to 3.46)**	**9.22 (5.67 to 15.0)**
No/unknown	Reference	Reference	Reference

Reference for all models: Low Morbidity group.

Most Hodgkin lymphoma patients received chemotherapy; therefore, chemotherapy was not included in the models.

Bold indicates statistical significance at *P* < .05.

#### Clinical factors

Survivors receiving radiation had higher odds of being in the Multiple Conditions (OR = 2.31, 95% CI = 1.41 to 3.78) and Thyroid (OR = 2.81, 95% CI = 2.20 to 3.58) than the Low Morbidity groups compared to survivors who did not receive radiation (Table 3). Survivors with a HCT had greater odds of being in the Multiple Conditions group (OR = 9.50, 95% CI = 5.82 to 15.5), Cardiovascular (OR = 3.82, 95% CI = 2.96 to 4.93), and Thyroid (OR = 2.86, 95% CI = 2.12 to 3.85) groups than those who did not receive HCT.

### Multivariable Cox proportional hazards regression models

**Figure 3.**
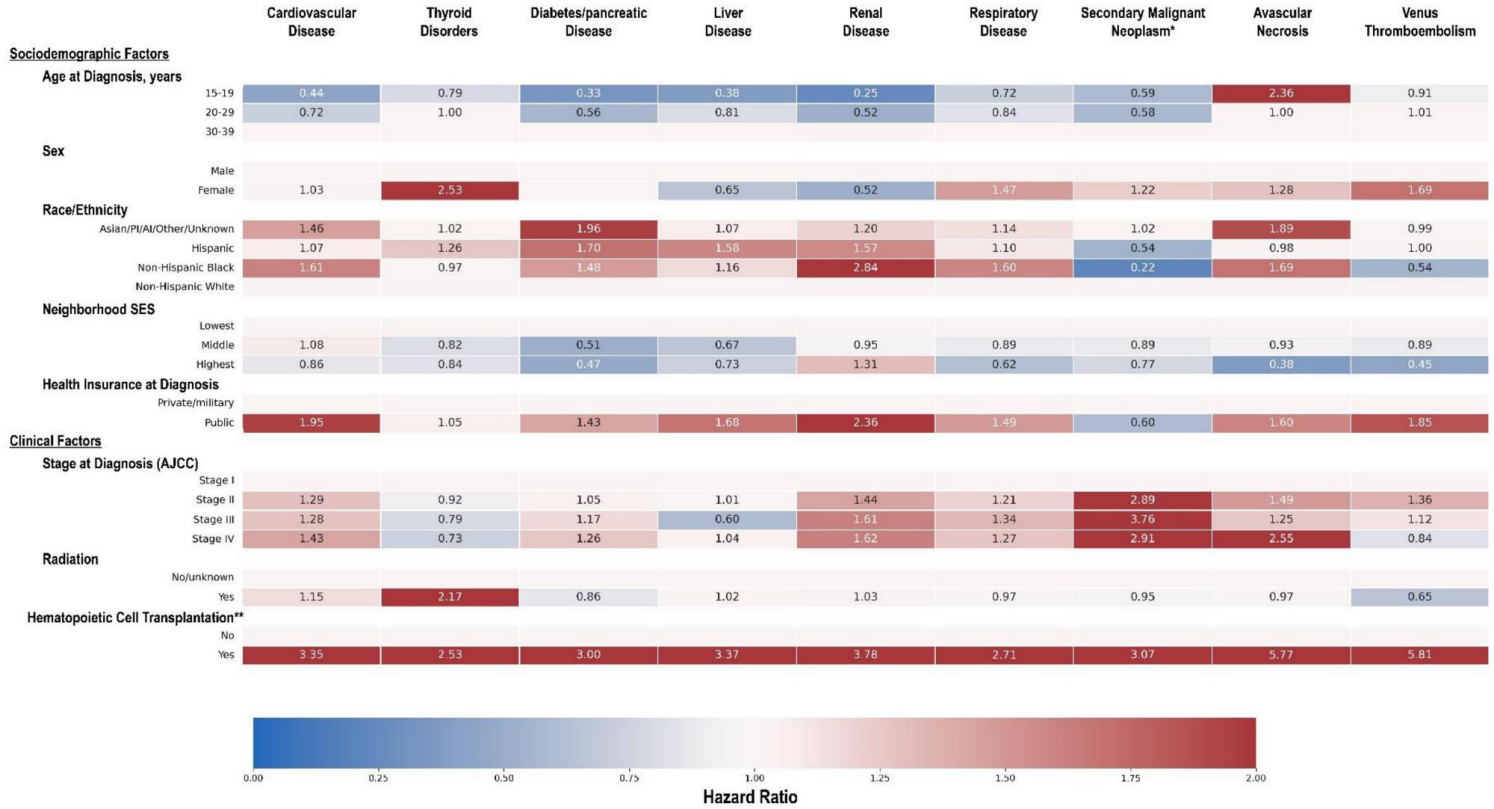
Heat map of statistically significant^±^ hazard ratios from Cox proportional hazards models for associations between sociodemographic and clinical factors and each late effect among 2-year survivors of adolescent and young adult Hodgkin lymphoma. SES, socioeconomic status; RT, radiation therapy; HCT, hematopoietic cell transplantation. ^±^Hazard ratios that met the threshold of statistical significance at *P* < .05. **Time-dependent variable.

When individual late effects were examined, younger survivors aged 15-19 years at diagnosis had a lower hazard of most late effects compared to those aged 30-39 at diagnosis (Figure 3; Table S2). Survivors aged 20-29 years had a lower risk for cardiovascular, diabetes, and renal late effects than those 30-39 years at diagnosis. Female survivors had elevated risks of thyroid and respiratory conditions, but lower risks of liver and renal late effects, compared to males. Cardiovascular, diabetes/pancreatic, liver, renal, and respiratory late effects were more common among multiple minoritized race/ethnicity survivors compared to non-Hispanic White survivors. Similarly, survivors with public (vs private/military insurance) also had elevated risks of these 5 conditions. Clinical factors included higher risks of second cancers for patients diagnosed at Stage II–IV (vs Stage I). Survivors who had a HCT had elevated hazards of all late effects compared to those who did not undergo HCT.

## Discussion

In this large population-based study, we investigated the incidence and clustering of late effects among AYA cHL survivors who were treated during a period of widespread use of risk-adapted therapy aimed at minimizing cumulative chemotherapy and radiation doses to healthy tissue. We found that for AYA cHL cancer survivors in California and Utah diagnosed between 2006 and 2018, most experienced no late effects that required an emergency department visit, hospitalization, or ambulatory surgery >2 years after cHL diagnosis, but that certain subgroups of survivors did experience late effects. The late effects that occurred most commonly were cardiovascular, thyroid, and respiratory disorders. When we examined late effects clusters, we found that a small proportion (2%) of survivors had a high probability of being in the Multiple Conditions group, including cardiovascular disease, diabetes/pancreatic disease, thyroid disease, and renal disease, while higher proportions of survivors were at a moderate probability of being in the Thyroid and Cardiovascular groups (13% and 8%, respectively). Our study provides novel insights into the incidence of late effects among cHL cancer survivors in a recently treated cohort, late effects that may cluster together, and risk factors for certain late effects.

We found that survivors who were aged 30-39 years at their cancer diagnosis, had public insurance at diagnosis, or were treated with radiation therapy or HCT were more likely to be in the Multiple Conditions than the Low Morbidity group. Survivors with HCT also had a higher likelihood of being in both the Thyroid and Cardiovascular groups. Survivors who were older were more likely to be in the Cardiovascular group and those that received radiation were more likely to be in the Thyroid group when compared to the Low Morbidity group. Thus, AYA survivors in these specific demographic and treatment groups may have an increased risk for certain late effects and may benefit from evaluation of tailored surveillance for late effects and prevention strategies.

Using robust administrative data from hospitalizations, emergency department visits, and ambulatory surgery visits, our findings in an AYA population diagnosed from 2006 to 2018 suggest differences in the incidence of late effects from prior studies limited to pediatric cHL samples treated in earlier years. Analyses from CCSS showed that by 10 years from diagnosis, cumulative incidence of severe, life-threatening late effects for HL survivors, who had survived at least 5 years after cancer diagnosis, ranges from ∼10%-20% depending on treatment era.^5^, ^23^ In our cohort, onset of any severe late effect was 22.7% within a similar follow-up window but also includes patients who survived only 2-5 years after cancer diagnosis. While many notable differences between the 2 cohorts make comparisons difficult (eg CCSS use of self-reported data versus our use of a statewide healthcare utilization database), our results suggest that further investigation of differences between incidence of late effects in pediatric cHL survivors versus AYA survivors is warranted to understand the unique burden of late effects and survivorship needs of AYAs.

Our prior work in an earlier California cohort (diagnosed 1996-2012) found that 26% of AYA cHL survivors experienced at least one medical condition and 15% had 2 or more (median follow-up time of 9.5 years).^3^ This suggests that, similar to our analysis, AYA cHL survivors may have a higher risk of late effects than pediatric survivors, but that changes in treatment may be reducing these risks. For example, radiation strategies have significantly changed since the 1970s, with cHL patients now receiving lower doses, more targeted fields, and fewer patients that receive radiation at all.^5^ The current cohort consists of more patients treated with chemotherapy alone, with 57.1% of survivors having received no radiation, and showed radiation as a risk factor for being in the Multiple Conditions and Thyroid groups in our LCA model. Receipt of radiation was identified as a risk factor for thyroid disease, but it was not a risk factor for any other individual late effect. The association between radiation and thyroid disease, but not with other late effects, may reflect the decreasing risk of more modern radiation therapy approaches with lower doses and smaller fields, which have been predicted to result in less cardiotoxicity^24^ and decreased secondary breast cancer risk.^25^

Potential multimorbidity among pediatric cancer survivors has been well documented. Many of the risk factors such as receipt of radiation predispose survivors to multiple late effects, but there may be a more complicated relationship, with the occurrence of one late effect predisposing survivors to the development of other late effects. A large analysis of 7670 survivors from the French Childhood Cancer Survivors Study cohort found patients with SMN had increased risk of severe cardiac disease compared to survivors who did not experience SMN. This increased risk for severe cardiac disease persisted even after controlling for shared risk factors of both SMN and cardiovascular disease, including receipt of radiation and anthracycline doses.^11^ In our current LCA approach, the Multiple Conditions and Cardiovascular groups both had increased risk of cardiovascular disease and increased risk for SMN. Our findings demonstrate the need for additional studies to better understand these relationships among late effects.

Our study aligns with and enhances past studies by highlighting the associations between sociodemographic risk factors and risk of late effects, including multimorbidity clustering. While neighborhood SES was not associated with clustering of late effects in our LCA, our study showed that patients residing in higher SES neighborhoods had a lower likelihood of diabetes/pancreatic, cardiovascular, and respiratory disease than survivors in the lowest SES neighborhoods. Additionally, our results showed that survivors with public insurance at diagnosis not only had a higher likelihood of many individual late effects (cardiovascular, liver, renal, and respiratory), but also had a higher likelihood of being in the Multiple Conditions versus Low Morbidity LCA group. These findings are similar to our prior work in an older California cHL survivor cohort, illustrating the multifaceted complexity associated with insurance and financial barriers for AYA cancer patients.^3^ The implementation of the Patient Protection and Affordable Care Act (ACA) may improve outcomes by enhancing AYA cancer survivors’ access to health insurance and reducing financial burden.^26^^,^^27^ ACA provisions included expanded dependent coverage to age 26 years, and additional avenues to insurance coverage via Marketplace plans (2014) and Medicaid expansion.^7^ We had limited follow-up time to investigate the potential impact of both Medicaid and dependent coverage expansion on late effect incidence and clustering, which should be a focus of future research.

Our study has certain limitations. While this population of AYA cHL survivors from 2 states provided a large sample size for analysis, our focus on emergency department, hospitalization, and ambulatory surgery records to ascertain late effects likely underestimates the true burden of late effects by not including those diagnosed and treated solely in outpatient settings, thus with potentially mild or moderate late effects. Our cohort does not include federal hospitals, which represent a small proportion of U.S. Hospitals (3.4% according to the American Hospital Association Annual Survey)^28^; however, this may result in underrepresentation of specific populations such as veterans and American Indian and Alaska Natives. Additionally, linkage required a valid SSN, which may have slightly limited our sample for certain demographic groups, affecting our external validity. We also did not have records of doses of specific chemotherapeutic agents or radiation doses, fields, or modalities. While we know in general that risk-adapted treatment with modern radiation techniques was standard of care nationally during the years of our study, the lack of detailed treatment information limits our ability to explore the nuances of more modern therapy on the development of late effects.

In this large population from 2 states with demographic and geographic variability, we found that among AYA cHL patients who had survived for at least 2 years, nearly 80% of survivors belonged to the Low Morbidity group and the cumulative incidence of each late effect was less than 10% at 10 years after diagnosis. However, our study identified groups of survivors with an increased probability of being in the Multiple Conditions, Thyroid, and Cardiovascular groups. In particular, receipt of radiation therapy or HCT and sociodemographic factors were associated with increased risks of being in these LCA groups. This work points to several areas of future research, including efforts to understand the reasons for clustering of late effects, which could differ according to treatment location (eg adult vs pediatric, community vs academic), and to evaluate interventions to alleviate disparities in the incidence of late effects for specific populations.

## Supplementary Material

pkaf094_Supplementary_Data

## Data Availability

Data that support the findings in this study are available from the original data custodians and not from the study authors. The data from California that support the findings of this study are available from the *California Cancer Registry*. Access to the data is granted through an application process by the management or data custodians. Access to data from the *UPDB* may be obtained through application by researchers to the Utah Resource for Genetic and Epidemiologic Research (RGE) at https://rge.utah.edu, which oversees access to the UPDB. Data from *Utah Cancer Registr*y are not publicly available due to privacy restrictions. Access may be granted through an application process reviewed by a data oversight committee.
