# Supplementary material for: Identifying clustering in patterns of late effects among survivors of adolescent and young adult Hodgkin lymphoma
Source: JNCI Cancer Spectr. 2025 Oct 1;9(6):pkaf094. doi: 10.1093/jncics/pkaf094 (PMC12628312; doi:10.1093/jncics/pkaf094)

**Supplementary Material**

**Supplementary Tables**

**Table S1: Cumulative incidence of late effects among 2-year survivors of adolescent and young adult Hodgkin lymphoma at 5- and 10-years after cancer diagnosis**

| **Late effect** | **5-year (95% CI)** | **10-year (95% CI)** |
| --- | --- | --- |
| Cardiovascular disease | 4.13 (3.56, 4.76) | 8.02 (7.13, 8.99) |
| Thyroid disorders | 3.36 (2.84, 3.94) | 9.30 (8.28, 10.38) |
| Diabetes/pancreatic disease | 2.06 (1.67, 2.53) | 4.32 (3.64, 5.09) |
| Liver disease | 1.46 (1.13, 1.85) | 3.37 (2.78, 4.05) |
| Renal disease | 0.70 (0.48, 0.99) | 1.85 (1.40, 2.39) |
| Respiratory disease | 5.53 (4.86, 6.25) | 10.80 (9.75, 11.91) |
| Secondary malignant neoplasm | 1.14 (0.86, 1.48) | 2.48 (1.98, 3.07) |
| Avascular necrosis | 0.36 (0.21, 0.58) | 0.91 (0.61, 1.32) |
| Venous thromboembolism | 1.03 (0.74, 1.39) | 1.76 (1.33, 2.29) |

**Table S2: Multivariable adjusted hazard ratios (HR) and associated 95% confidence intervals (95% CI) for associations between sociodemographic and clinical factors with late effects among 2-year survivors of adolescent and young adult Hodgkin lymphoma, 2006-2018**

|  | **Cardio-vascular disease** | **Thyroid disorders** | **Diabetes/ pancreatic disease** | **Liver disease** | **Renal disease** | **Respiratory disease** | **Secondary malignant neoplasm*** | **Avascular necrosis** | **Venous thrombo-embolism** |
| --- | --- | --- | --- | --- | --- | --- | --- | --- | --- |
|  | **HR**  **(95% CI)** | **HR**  **(95% CI)** | **HR**  **(95% CI)** | **HR**  **(95% CI)** | **HR**  **(95% CI)** | **HR**  **(95% CI)** | **HR**  **(95% CI)** | **HR**  **(95% CI)** | **HR**  **(95% CI)** |
| **Sociodemographic Factors** |  |  |  |  |  |  |  |  |  |
| **Age at diagnosis, years** |  |  |  |  |  |  |  |  |  |
| 15-19 | **0.44**  **(0.30, 0.64)** | 0.79  (0.57, 1.09) | **0.33**  **(0.19, 0.56)** | **0.38**  **(0.20, 0.70)** | **0.25**  **(0.10, 0.64)** | **0.72**  **(0.53, 0.98)** | 0.59  (0.32, 1.08) | 2.36  (0.87, 6.40) | 0.91  (0.41, 2.01) |
| 20-29 | **0.72**  **(0.57, 0.91)** | 1.00  (0.79, 1.28) | **0.56**  **(0.41, 0.77)** | 0.81  (0.56, 1.17) | **0.52**  **(0.32, 0.85)** | 0.84  (0.68, 1.05) | **0.58**  **(0.38, 0.88)** | 1.00  (0.39, 2.55) | 1.01  (0.56, 1.79) |
| 30-39 | Reference | Reference | Reference | Reference | Reference | Reference | Reference | Reference | Reference |
| **Sex** |  |  |  |  |  |  |  |  |  |
| Female | 1.03  (0.83, 1.29) | **2.53**  **(2.00, 3.20)** | ****** | **0.65**  **(0.46, 0.92)** | **0.52**  **(0.31, 0.87)** | **1.47**  **(1.20, 1.79)** | 1.22  (0.82, 1.81) | 1.28  (0.61, 2.68) | 1.69  (0.98, 2.89) |
| Male | Reference | Reference |  | Reference | Reference | Reference | Reference | Reference | Reference |
| **Race/ethnicity** |  |  |  |  |  |  |  |  |  |
| Asian or Pacific Islander, other, unknown | **1.46**  **(1.03, 2.09)** | 1.02  (0.69, 1.50) | **1.96**  **(1.16, 3.32)** | 1.07  (0.54, 2.13) | 1.20  (0.48, 3.00) | 1.14  (0.80, 1.63) | 1.02  (0.55, 1.87) | 1.89  (0.58, 6.16) | 0.99  (0.33, 2.91) |
| Hispanic | 1.07  (0.81, 1.41) | 1.26  (0.96, 1.64) | **1.70**  **(1.18, 2.46)** | **1.58**  **(1.06, 2.37)** | 1.57  (0.87, 2.81) | 1.10  (0.86, 1.41) | **0.54**  **(0.31, 0.95)** | 0.98  (0.37, 2.57) | 1.00  (0.55, 1.80) |
| Non-Hispanic Black | **1.61**  **(1.09, 2.40)** | 0.97  (0.60, 1.57) | 1.48  (0.81, 2.69) | 1.16 (0.57, 2.39) | **2.84**  **(1.32, 6.10)** | **1.60**  **(1.13, 2.26)** | **0.22**  **(0.05, 0.90)** | 1.69  (0.48, 5.98) | 0.54  (0.19, 1.54) |
| Non-Hispanic White | Reference | Reference | Reference | Reference | Reference | Reference | Reference | Reference | Reference |
| **Neighborhood SES** |  |  |  |  |  |  |  |  |  |
| Highest | 0.86  (0.63, 1.18) | 0.84  (0.62, 1.14) | **0.47**  **(0.31, 0.72)** | 0.73  (0.46, 1.15) | 1.31  (0.69, 2.48) | **0.62**  **(0.47, 0.83)** | 0.77  (0.44, 1.33) | 0.38  (0.12, 1.19) | 0.45  (0.20, 1.05) |
| Middle | 1.08  (0.82, 1.42) | 0.82  (0.62, 1.09) | **0.51**  **(0.35, 0.76)** | 0.67  (0.44, 1.03) | 0.95  (0.52, 1.75) | 0.89  (0.70, 1.14) | 0.89  (0.52, 1.51) | 0.93  (0.41, 2.12) | 0.89  (0.50, 1.57) |
| Lowest | Reference | Reference | Reference | Reference | Reference | Reference | Reference | Reference | Reference |
| **Health insurance** |  |  |  |  |  |  |  |  |  |
| Private/military | Reference | Reference | Reference | Reference | Reference | Reference | Reference | Reference | Reference |
| Public | **1.95**  **(1.52, 2.50)** | **1.05**  **(0.80, 1.38)** | **1.43**  **(1.00, 2.05)** | **1.68**  **(1.13, 2.51)** | **2.36**  **(1.35, 4.13)** | **1.49**  **(1.18, 1.88)** | 0.60  (0.34, 1.07) | 1.60  (0.77, 3.33) | 1.85  (0.99, 3.47) |
| **Clinical Factors** |  |  |  |  |  |  |  |  |  |
| **Stage at diagnosis (AJCC)** |  |  |  |  |  |  |  |  |  |
| Stage I | Reference | Reference | Reference | Reference | Reference | Reference | Reference | Reference | Reference |
| Stage II | 1.29  (0.89, 1.88) | 0.92  (0.67, 1.26) | 1.05  (0.64, 1.71) | 1.01  (0.61, 1.67) | 1.44  (0.58, 3.58) | 1.21  (0.86, 1.69) | **2.89**  **(1.15, 7.29)** | 1.49  (0.30, 7.41) | 0.54  (0.19, 1.54) |
| Stage III | 1.28  (0.84, 1.95) | 0.79  (0.53, 1.17) | 1.17  (0.69, 1.98) | 0.60  (0.32, 1.13) | 1.61  (0.63, 4.12) | 1.34  (0.93, 1.95) | **3.76**  **(1.43, 9.88)** | 1.25  (0.22, 6.93) | 1.00  (0.55, 1.80) |
| Stage IV | 1.43  (0.93, 2.20) | 0.73  (0.47, 1.12) | 1.26  (0.73, 2.18) | 1.04  (0.56, 1.92) | 1.62  (0.60, 4.43) | 1.27  (0.85, 1.88) | **2.91**  **(1.05, 8.10)** | 2.55  (0.48,13.44) | 0.99  (0.33, 2.91) |
| **Radiation** |  |  |  |  |  |  |  |  |  |
| Yes | 1.15  (0.91, 1.45) | **2.17**  **(1.74, 2.70)** | 0.86  (0.62, 1.21) | 1.02  (0.71, 1.48) | 1.03  (0.59, 1.79) | 0.97  (0.78, 1.20) | 0.95  (0.62, 1.44) | 0.97  (0.42, 2.23) | 0.65  (0.36, 1.16) |
| No/unknown | Reference | Reference | Reference | Reference | Reference | Reference | Reference | Reference | Reference |
| **Hematopoietic cell transplantation**** |  |  |  |  |  |  |  |  |  |
| **Yes** | **3.35**  **(2.63, 4.27)** | **2.53**  **(1.96, 3.26)** | **3.00**  **(2.16, 4.17)** | **3.37**  **(2.31, 4.91)** | **3.78**  **(2.24, 6.38)** | **2.71**  **(2.17, 3.38)** | **3.07**  **(2.03, 4.65)** | **5.77**  **(2.61, 12.76)** | **5.81**  **(3.45, 9.79)** |
| No/unknown | Reference | Reference | Reference | Reference | Reference | Reference | Reference | Reference | Reference |

Most Hodgkin Lymphoma patients received chemotherapy; therefore, chemotherapy was not included in the models. Data for other/unknown race/ethnicity not presented due to the small number of patients in this category. Sex violated the PH assumption and so was stratified in the diabetes/pancreatic disease model.

*Ascertainment of secondary cancer started 60 days after cancer diagnosis, with secondary cancers occurring <2 years considered as outcomes at the beginning of study follow-up. **Time-dependent variable.

**Supplementary Figures**

**Figure S1: Cohort Diagram**

AYA cHL diagnosed ages 15-39 years

2006-2018

1st primary

N= 5843

Exclude < 2 years follow-up: N=636

- Died < 2 years (includes Autopsy/Discharge): N= 152

- Missing cancer dx month or follow-up month: N= 28

- Follow-up time < 2 years: N= 456

AYA cHL diagnosed ages 15-39 years

2006-2018

1st primary & ≥2 years of follow-up

N= 4648

**Exclusions:** Concurrent cancer diagnosis (within 60 days) or HIV/AIDS: N= 13

**Exclude Unknown/Invalid SSN (unlinkable):** N= 559

**ANALYSIS COHORT**

AYA cHL diagnosed ages 15-39 years

2006-2018

1st primary & ≥2 years of follow-up

N= 4635

**Figure S2: Distribution of late effects within each latent class group among 2-year survivors of adolescent and young adult Hodgkin lymphoma**
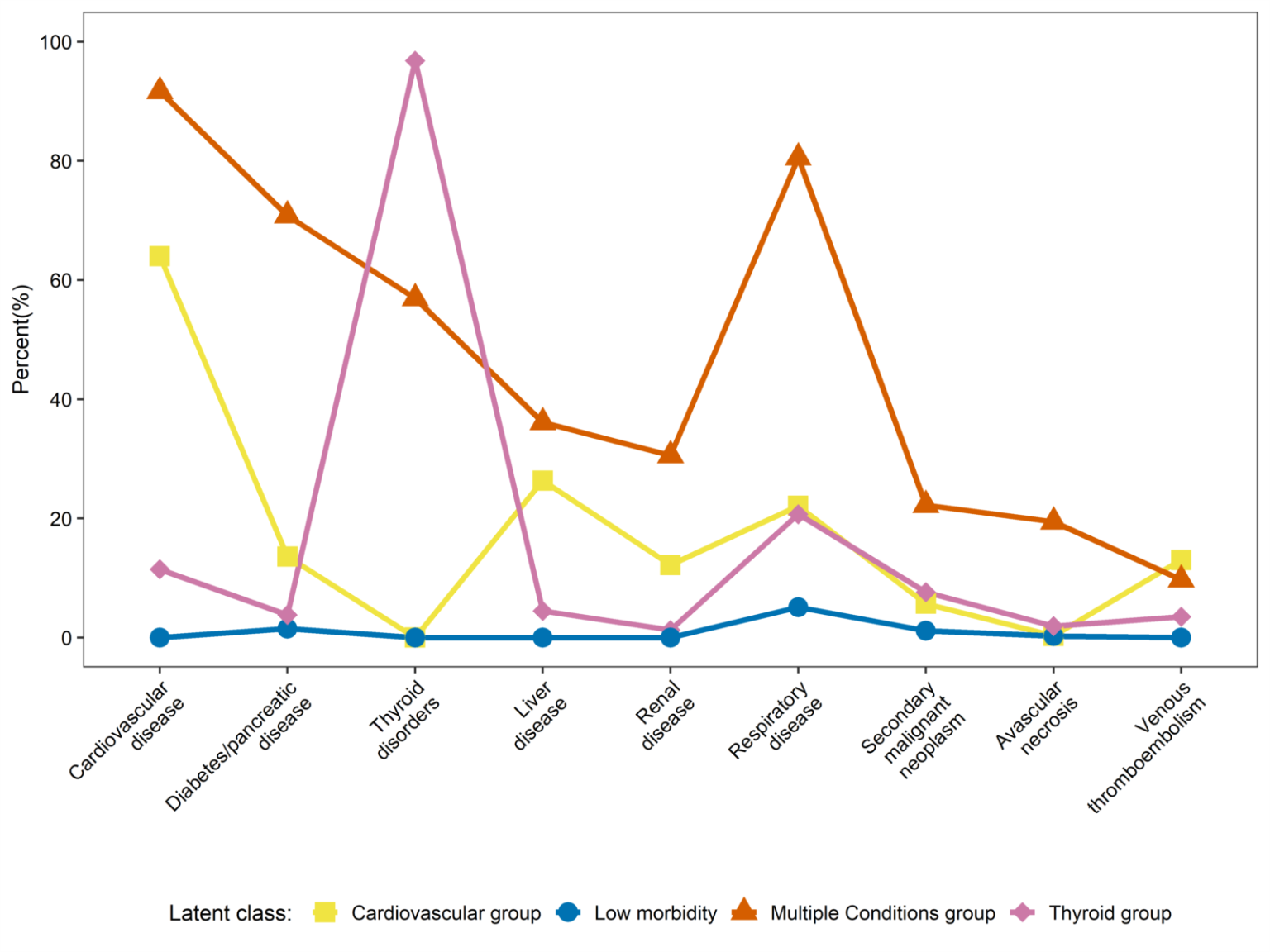

Supplement: pkaf094_Supplementary_Data [file pkaf094_supplementary_data.docx]
